# Disease monitoring programs of rare genetic diseases: transparent data sharing between academic and commercial stakeholders

**DOI:** 10.1186/s13023-021-01687-7

**Published:** 2021-03-20

**Authors:** Hanns Lochmüller, Antonio Nino Ramirez, Emil Kakkis

**Affiliations:** 1 Children’s Hospital of Eastern Ontario Research Institute, Ottawa, Canada; 2 Division of Neurology, Department of Medicine, The Ottawa Hospital, Ottawa, Canada; 3 Brain and Mind Research Institute, University of Ottawa, Ottawa, Canada; 4 Ultragenyx Pharmaceutical Inc., Novato, CA USA

**Keywords:** Orphan drugs, Registry, Registries, Data sharing, Disease monitoring program, DMP, GNE myopathy, GNE myopathy disease monitoring program, GNEM-DMP

## Abstract

It has recently been suggested that registries for rare neuromuscular diseases should be formed and governed exclusively by physicians and patients in an effort to limit conflicts of interest. Enacting such an approach would not only be challenging logistically and financially, but it would also exclude the involvement of sponsors, who are an integral component of drug development within the current compliance framework. Therefore, as an alternative to traditional registries, we propose the use of a better collaborative model for post-marketing follow-up that includes all stakeholders. We developed the concept of Disease Monitoring Programs (DMPs), which are designed to monitor disease manifestations over a 10-year period whether on a sponsored drug or not, and ensure consistent collection, ownership sharing and governance of data.

Hollak et al. have provided a provocative argument that registries for orphan drugs designed by pharmaceutical companies are tainted by conflicts of interest since they are conducted by the sponsors of a product [1]. This is not an entirely new concept, as some early examples related to lysosomal storage diseases may demonstrate, e.g., competing product-specific registries by different companies, poor data quality, insufficient recruitment, lack of transparency and lack of data sharing. We do appreciate and agree that the proprietary approach taken by many sponsors in conducting these registries does not advance the understanding and treatment of rare diseases as well as it should, and could be improved. The International Rare Disease Research Consortium (IRDiRC) representing public and private research funders and patient organizations from around the world, provided guidance and best-practice examples to a more effective, transparent, and balanced approach, including governance, data sharing, and data linkage models [2, 3]. We disagree with the authors’ proposed solution of registries formed and governed exclusively by physicians and patients for several reasons. While Hollak et al. implied biased decisions and lack of data disclosure, the substitution of the academic world and patient groups to conduct such registries would not solve the problem and has rarely been successfully done in an effective and appropriately compliant manner, due not only to the millions of Euros required but also to the numerous diverse professionals required to conduct such studies that are rarely present in most academic institutions. The pharmaceutical industry sponsored registries are required to be designed and conducted by sponsors as an integral part of the whole drug development process within the legal regulatory and compliance framework exerted by US Food and Drug Administration, European Medicines Agency, and other regulators to ensure that the studies abide by the requirements that society imposes on drug developers to maintain a license to operate. To extract the pharmaceutical sponsors from registries for rare diseases would not solve the problem, nor would it end the need for the pharmaceutical sponsors to conduct the registries to meet their regulatory requirements. We would propose instead a more efficient and transparent collaboration between regulatory agencies, academia, industry, clinicians, and patient advocacy groups to optimize the long-term follow-up of disease manifestations and product outcomes.

We advocate for a new model of registries where collaboration exists in which the pharmaceutical sponsor partners with a major physician organization on the ownership and management of data and forms a steering committee of stakeholders to manage the evaluation and processing of data and requests for evaluation. We call these studies Disease Monitoring Programs (DMP) to distinguish them from traditional registries. The DMPs are intended to monitor disease manifestations over a 10-year period in patients both on a sponsored drug, or on other treatment, or not treated at all, in a comprehensive GCP-monitored format where all measurements, tests, patient travel and physician effort is covered by the Sponsoring company, to assure the data are collected consistently as scheduled and not missing as happens in some academic programs. The GNE Myopathy Disease Monitoring Program (GNEM-DMP) is an example of this DMP partnership between TREAT-NMD and Ultragenyx Pharmaceutical Inc. (USA), with shared ownership of data governed by a collaboration agreement [4, 5]. The GNEM-DMP began before aceneuramic acid extended release was submitted for a marketing authorization and was used to collect natural history data across a number of countries but would also have potentially collected data for patients on a drug in the post-marketing setting. The use of the data in the GNEM-DMP was governed by a team of researchers, physicians, and patient representatives from US, UK, Israel, and Japan who constitute the steering committee. The program aims to improve the medical knowledge of GNE myopathy and can be a model for future registries for rare diseases to gain knowledge on the natural history of the disease and the effect of all available treatments. In this specific program, the phase 3 study of the sponsored drug failed, and the dataset, conduct, and ownership of the DMP data was continued by TREAT-NMD with the data available to others as was agreed by the collaboration agreement. Any academic or industry researcher can request access to data, perform independent analysis, and publish findings free from influence of commercial sponsors once approved by the steering committee. We think this structured collaborative approach of a DMP can achieve a more productive and transparent outcome, taking the skills and contributions of each party to assure the greater interests of the patient community and society are met.

From our experience in rare and neuromuscular disease registries, successful public–private partnership models have been constructed in different ways. Ideally, these models should facilitate research at different stages of drug development and application, be flexible enough to adopt new research questions and new contributors, and have a long-term sustainability plan [6–8].

## Data Availability

Not applicable.

## Abbreviations

DMP: Disease monitoring program
GNEM-DMP: GNE myopathy disease monitoring program
NMD: Neuromuscular disease
